# Large right ventricular hydatid cyst in a child: a case report

**DOI:** 10.1186/s43044-023-00386-x

**Published:** 2023-07-22

**Authors:** Kourosh Vahidshahi, Tahmineh Tahouri, Farzaneh Farahmandi, Manouchehr Hekmat

**Affiliations:** 1 School of Medicine, Shahid Modarres Educational Hospital, Shahid Beheshti University of Medical Sciences, Tehran, Iran; 2 Department of Pediatric Cardiology, School of Medicine, Shahid Modarres Educational Hospital, Shahid Beheshti University of Medical Science, Intersection of Saadat Abad and Yadegar Imam Highway, Tehran, Iran

**Keywords:** Hydatid cyst, Echinococcosis, Pulmonary hydatid cyst, Heart

## Abstract

**Background:**

Cystic Hydatid disease is a parasitic infection with a worldwide distribution. It is caused by the larval stages of a species of tapeworms known as *Echinococcus granulosus*. Even in endemic areas; Cardiac involvement by hydatidiosis is very rare and has atypical presentations as well as localization which make it undiagnosed in about 10% of cases. The left ventricle is the most Common chamber involved by the hydatid cyst and isolated involvement of the right ventricle is very rare, especially in children. The aim of the present study was to describe hydatid cardiac cyst of the right ventricle of a child.

**Case presentation:**

We present a rare case of an 8 year-old boy, living in a rural area, who was diagnosed with a cardiac hydatid cyst in the right ventricle. He also had multiple pulmonary hydatid cysts and presented with dyspnea, cough and atypical chest pain. The patient underwent surgery for the resection of pulmonary cysts and, subsequently, cardiac hydatid cyst. The outcome was favorable seven weeks after surgery and there was no clinical and echocardiographic recurrence.

**Conclusion:**

Cardiac Echinococcosis must be suspected in endemic areas, diagnosed with appropriate imaging techniques, and treated appropriately.

**Supplementary Information:**

The online version contains supplementary material available at 10.1186/s43044-023-00386-x.

## Background

Cystic Hydatid disease is a parasitic infection with a worldwide distribution. Hyper endemic areas for this disease including the Middle East, South America, Australia, New Zeeland, Asia, Africa and the Mediterranean coast. *Echinococcus granulosus*, which is also known as “hydatid worm” or “canine tapeworm”, is a species of tapeworm and it is the main cause of cystic hydatid disease. Human infection occurs through eating food or water contaminated with Echinococcal embryonated eggs, which are often present in the faeces of animal hosts of Echinococcus (dogs and other canines). Inside the human small intestine, the embryo hatches and penetrates the intestinal wall and reaches various organs through the blood stream [1]. In the organs such as liver (50–70%), lungs (20–30%), muscles (5%), bones (3%), kidneys (2%), spleen (1%), brain (1%) and rarely the heart (0.5–2%), Echinococcus larval stages develop into hydatid cysts [1–3]. Even in endemic areas, cardiac involvement by hydatidiosis is very rare. There are two possible explanations for this: firstly, cardiac contractions may prevent the cyst from implanting in the heart, and secondly, the embryo is usually filtered through the liver and lungs [4]. The left ventricle is the most common cardiac chamber affected by *Echinococcus granulosus*. Cardiac hydatid cyst has a long incubation period and grows slowly (about 1 cm per year in children) and there are no clinical symptoms unless the cardiac cyst is large enough. Symptoms, if present, are usually atypical and depend on the location, age, size as well as integrity of the cyst. Palpitations, chest pain, cough and dyspnea are the most common symptoms of cardiac hydatid cyst. Accordingly, diagnosis of cardiac hydatid cyst is difficult and requires high clinical suspicion [4, 5]. In addition, cardiac hydatid cyst may have serious and fatal complications, including anaphylactic shock secondary to sub-endocardial rupture, arrhythmia, systemic or pulmonary embolization, pulmonary hypertension and sudden cardiac death [4, 6]. Therefore, timely diagnosis of cardiac hydatid cyst is very crucial. However, few studies have been conducted on cardiac hydatid cyst in children and there is relatively little information about the symptoms of this disease and how to diagnose and treat it. The aim of the present study was to describe a case of hydatid cyst of the right ventricle.

## Case presentation

An eight-year-old boy living in a rural area presented to the Emergency Department of Mofid Children’s Hospital, Tehran, Iran, with dyspnea, cough and atypical chest pain from one month ago. The patient was otherwise healthy and had received standard immunization (including BCG). There was no relevant familial history and the patient had normal growth and development and the patient was otherwise healthy.

At the time of admission, his blood pressure and pulse rate were 105/65 mmHg and 98 beats per minute respectively. He had no respiratory distress and jugular veins were not prominent. Auscultation revealed coarse crackles in both lung and normal heart sounds. Physical examinations of other body systems were normal.

**Fig. 1:**
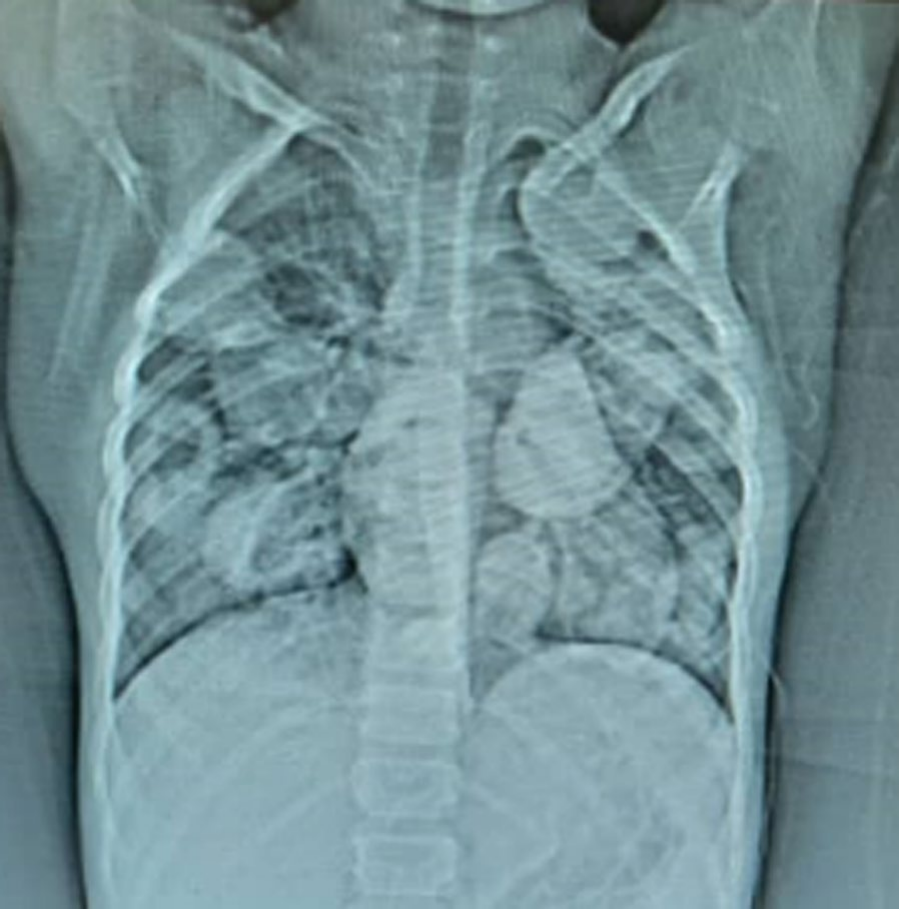
Plain chest X-ray at AP view shows multiple bilateral areas of consolidation

**Fig. 2:**
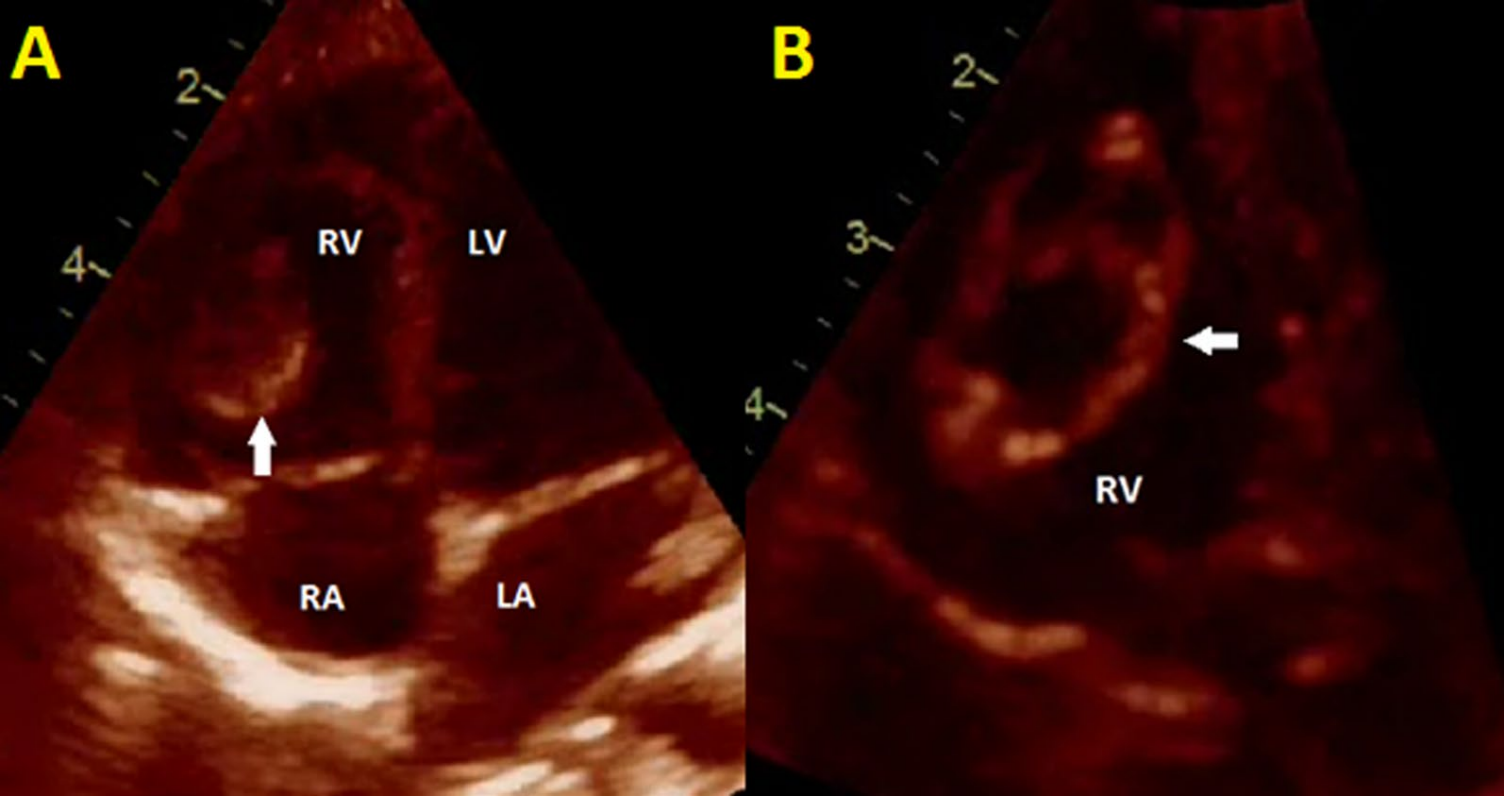
Trans-thoracic echocardiogram at four chamber view **A** shows large cystic lesion (white Arrows) with adhesion to the right ventricular free wall. Tilting the probe into posterior **B** reveals a large multivesicle cyst in the right ventricle. *RA* Right atrium; *RV* Right ventricle; *LA* Left atrium; *LV* Left ventricle

The Electrocardiogram showed a normal sinus rhythm with a normal axis. Laboratory investigations revealed a mild leukocytosis with mild eosinophilia (white blood cell count of 15,900/mm^3^ with differential count of neutrophil: 61%, lymphocyte: 29%, eosinophil: 7% and monocyte: 3%), erythrocyte sedimentation rate of 64 mm/hour and C-reactive protein of 2+positive. The alanine aminotransferase (ALT) and aspartate aminotransferase (ALT) were within normal ranges. Plain chest X-ray, multiple bilateral areas of rounded consolidation were obvious; the cardio-thoracic ratio was normal (Fig. 1). Subsequently, a Thoraco-abdominal computed tomography was performed, which demonstrated multiple cystic lesions in both lungs. Trans-thoracic echocardiography revealed a relatively large echo-lucent septated cystic lesion (measured 2.1*1.8 mm) with a thin outer wall and adhesion to the right ventricular free wall (Fig. 2 and movie Additional file 1: Clip S1). There was no right ventricular obstruction and the cardiac function was normal. The abdominal as well as the brain computed tomography scan were normal.

Upon the computed tomography scan findings, the patient’s demographic origin and a positive.

Hem-agglutinin test for hydatid disease, the diagnosis of hydatid cyst was established. Accordingly, after a 5 day oral Albendazole course, the patient underwent surgery for resection of the lung cysts via bilateral thoracotomy.

**Fig. 3:**
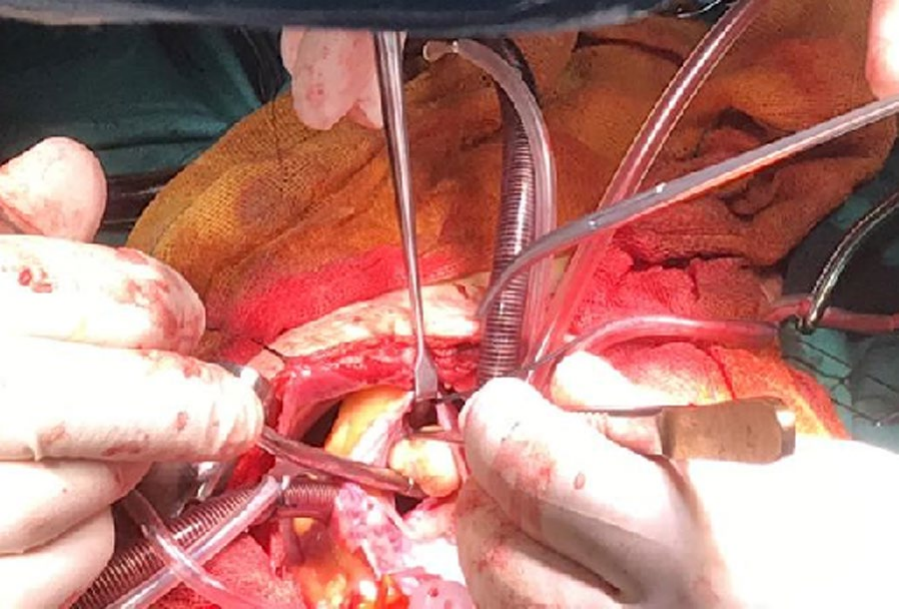
Intraoperative view. The cyst is apparent within the right ventricle with attachment to the right ventricular papillary muscle

Thereafter, the patient was referred to Shahid Modarres hospital, Tehran, Iran, which is a tertiary center of pediatric cardiac surgery for excision of the cardiac cyst. The patient underwent elective cardiac surgery through median sternotomy and cardio-pulmonary bypass (CPB). A small incision on the right atrium was made. In the examination through the tricuspid valve, the cyst was apparent within the right ventricle with attachment to the right ventricular papillary muscle (Fig. 3). Intra-operatively, in order to prevent systemic contamination, the contents of the hydatid cyst was aspirated and the cavity of the cyst was washed with saline solution and then the cyst membranes were removed. The diagnosis of hydatid cyst was confirmed in pathologic examination. Medical therapy with Albendazole (15 mg/kg) for 3 months started. The post-operative course was uneventful and he was discharged on the seventh day. The outcome was favorable seven weeks after surgery and there was no clinical and echocardiographic recurrence.

## Discussion

Cardiac involvement is very rare and consists of only 0.5–2% of all locations involved by cystic hydatid disease. The possible cause of its rarity is the filtration of embryos through the lung and the liver [4]; however, a small number of embryos may bypass the vascular bed of the lungs and liver and reach the myocardium via the coronary circulation, especially the left anterior descending artery [4, 7]. The other probable mechanism for cardiac hydatid cyst is involvement via the pulmonary veins [3]. Moreover, the ingested larvae can also involve the right side of the heart through the superior vena cava and thoracic duct [8]. Anyhow, the left ventricle is the most common chamber involved by the hydatid cyst (60%) and involvement of the right ventricle has been reported in 15%, the interventricular septum in 9%, the left atrium in 8%, the right atrium in 4%, and the interatrial septum in 2% of cases. Involvement of the pericardium by hydatid cyst is very rare [7]. The possible reason why left ventricle involvement is more common than the right ventricle is the fact that the left coronary artery is more dominant; on the other hand, the myocardial mass of the left ventricle is greater than the right ventricle, which provides a suitable environment for the development of the larvae [2]. However, the present case is not unique. Reviewing the literature demonstrated that less than 15 cases of isolated right ventricular hydatid Cysts (without left heart involvement) in children have been reported to date.

Cardiac hydatid cyst has atypical presentations which make it undiagnosed in about 10% of cases. Indeed, clinical symptoms of cardiac hydatid cyst range from asymptomatic to severe heart failure and other life threatening events [9]. The number, the size, the location and the revolutionary stage of the cyst, can all play a role in clinical presentations and physical examination of the patient [10]. Symptoms if present are including chest pain (due to the compression of coronary arteries or acute pericarditis and intra-pericardial cyst rupture), palpitation (due to the irritation of the conduction system located in the inter-ventricular septum), atrio-ventricular block (due to the compression of the conduction fibers), dyspnea or syncope (due to the right ventricular or left ventricular outflow tract obstruction) and tamponade (due to the rupture of an intra-pericardial cyst) [3]. Al-Dairy et al. reported partial right ventricular outflow obstruction secondary to a right ventricular hydatid cyst in a 9-year-old girl, who was presented with exertional dyspnea [4]. Moreover, life threatening events such as pulmonary embolism, anaphylactic shock and malignant arrhythmia can also occur [4, 6]. It has been reported that in cases of right ventricle hydatid cysts, intracavitary rupture is more frequent and may lead to pulmonary embolization, pulmonary hypertension, and death [4, 6, 11]. Demirci et al. reported a case of sudden cardiac death in a 10 year-old girl due to a large hydatid cyst located in the right ventricular apex [12]. Çetin et al. also reported pulmonary hypertension secondary to right ventricular as well as both the pulmonary arteries [13]. Additionally, there are reported cases of cardiac hydatid cyst in which, the only clinical presentation was a cardiac murmur [14, 15]. In a case reported by Macedo et al., the only presentation of a right ventricular hydatid cyst in an 11 year-old boy was an ejection murmur in the left sternal border [14]. Therefore, in hydatid cysts located on the right side of the heart, timely diagnosis and treatment can help in preventing these unfortunate and fatal consequences.

Clinical signs and symptoms are the main diagnostic tools, especially in endemic areas. Modalities such as transthoracic echocardiography, Computed tomography and magnetic resonance imaging, are all helpful in diagnosing hydatid cysts [4, 6, 7]. The differential diagnoses of a cardiac hydatid cyst include cardiac blood cyst, cardiac thrombus and cardiac tumors. Cardiac thrombus often occurs in patients with underlying heart disease such as atrial fibrillation of reduced ventricular function and presents as a non-echogenic mass. Cardiac blood cyst is usually seen in newborns and mainly affects the atrioventricular valves [16, 17]. Cardiac hydatid cyst has a different MRI presentation. In T1 sequences, it is hypointense and while in T2 sequences, it is hyperintense. Moreover, the presence of calcification of the cyst wall, membrane detachment and daughter cyst, can be useful to distinguish cardiac hydatid cyst from the other cardiac cystic masses [17, 18]. The characteristic manifestations of hydatid cyst in MRI can be useful to distinguish it from a cardiac tumor. However, in the cases in which the hydatid mass becomes solid, differentiating it from a cardiac tumor could be difficult [2].

There is no specific WHO guideline for the management of cardiac Echinococcosis [19] and the treatment of cardiac hydatid cyst is usually made by a combination of medical as well as surgical management; in fact, surgery is the treatment of choice and Albendazole as a prophylactic treatment is recommended after surgery [14]. The duration of post-operative albendazole varies in different articles. In the case reported by Al-Dairy et al., the surgery for the excision of the right ventricular hydatid cyst was performed after a 5 day course of oral Albendazole; the surgery was done via median sternotomy. They also administered Albendazole for 12 weeks after the surgery [4]. Macedo et al. also performed surgery for the resection of the right ventricular hydatid cyst; they did not give a pre-operative, but they put the patient on albendazole for 4 months after the surgery [14]. In the case reported by Cetin et al., surgery for the removal of the right ventricular hydatid cyst as well as the pulmonary endarterectomy was done after a course of oral albendazole, which was continued for 6 months after the surgery [13]. Diaz-Menendez et al. retrospectively studied the management of 11 adult patients with cardiac hydatid cyst and the mean duration of anthelminthic therapy in their study was 27 months [19]. The perioperative mortality for the surgery of cardiac Echinoccucus has been reported about 20%. The surgery is performed via right side thoracotomy or median sternotomy and using cardio-pulmonary bypass, cross clamping of the aorta and moderate hypothermia. In order to prevent pulmonary embolization, the clamping of the pulmonary artery is crucial during the surgery for the excision of the right ventricular hydatid cyst. Before the cystectomy, aspiration of the cysts should be done for macroscopic as well as microscopic evaluation. The sterile cysts contain clear fluid and at least one protoscolex. For the inactive lesions, aspiration alone with suction is sufficient. In cases in which the hydatid cysts are ruptured, cyst sterilization by using hypertonic saline, formaline, cetimide, silver nitrate or iodine solutions should be done before the excision of the cysts to prevent dissemination of the infection. Regardless, infection and anaphylactic shock are possible during surgery. Reviewing the literature showed that there are reports of peri-operative anaphylactic shock in patients with cystic hydatid disease. The estimated incidence of intra-operative hydatid cyst related anaphylactic shock is about 0.2–3.3%. The pathophysiology of anaphylactic shock in these patients is related to the leakage of the antigenic contents of the hydatid cyst into the systemic circulation or the body cavities [20]. In the present case, in order to prevent systemic contamination and resulting anaphylactic shock during the surgery, the contents of the hydatid cyst was aspirated and the cavity of the cyst was washed with saline solution.

Another potential risk which could be encountered during the surgery of the right ventricular hydatid cyst is the pulmonary embolism. In order to prevent the cyst contents from draining into the pulmonary circulation, it is recommended to clamp the pulmonary artery before the hydatid cyst removal [10]. Nevertheless, the pulmonary cross clamping was not done during the surgery in the present case.

For 5 years after the surgery, echocardiography as well as serologic examination is recommended in order to recurrences detection [5].

## Conclusion

Involvement of the right side of the heart is a rare presentation of hydatiosis in children. The clinical course varies widely, and the presentation may be insidious with persistent potential risk of life-threatening events. For any patient presenting with echynococcosis, thoracic symptom discovery should lead to at least a cardiac ultrasonography to rule out a possible cardiac localization. Apart from the treatment of other hydatid cyst localization, cardiac cyst treatment is medical and surgical.

## Supplementary Information

**Additional file 1:** **Movie Clip S1:** Two dimensional Transthoracic echocardiography in four chamber view showed a large cystic lesion in right ventricle.

## Data Availability

The authors confirm that the data supporting the findings of this study are available within the article and its Additional file.
